# Atrial Fibrillation Predicts Long-Term Outcome after Transcatheter Edge-to-Edge Mitral Valve Repair by MitraClip Implantation

**DOI:** 10.3390/biom8040152

**Published:** 2018-11-19

**Authors:** Mirjam Keßler, Alexander Pott, Elnura Mammadova, Julia Seeger, Jochen Wöhrle, Wolfgang Rottbauer, Sinisa Markovic

**Affiliations:** Department of Internal Medicine II, University of Ulm, 89081 Ulm, Germany; mirjam.kessler@uniklinik-ulm.de (M.K.); Alexander.Pott@uniklinik-ulm.de (A.P.); Elnura.mammadova@uniklinik-ulm.de (E.M.); Julia.Seeger@uniklinik-ulm.de (J.S.); Jochen.Woehrle@uniklinik-ulm.de (J.W.); Wolfgang.Rottbauer@uniklinik-ulm.de (W.R.)

**Keywords:** MitraClip, atrial fibrillation, mortality, MACCE, outcome

## Abstract

*Background*: Atrial fibrillation is common in patients with mitral regurgitation (MR) and has a negative impact on the clinical outcome of patients with valvular heart disease. We aimed to evaluate the impact of pre-procedural atrial fibrillation on the long-term clinical outcomes of patients with MR undergoing transcatheter mitral valve repair by MitraClip implantation. *Methods*: We analysed 355 consecutive patients with and without atrial fibrillation with symptomatic, severe MR and inoperability or high surgical risk undergoing MitraClip implantation in a three-year follow-up. *Results*: In patients with pre-procedural atrial fibrillation undergoing MitraClip implantation, we found advanced age, higher baseline NT-pro-BNP levels, increased left atrial diameter, and higher rate of severe tricuspid regurgitation, compared to patients with sinus rhythm. In the three-year follow-up after MitraClip implantation, mortality and major adverse cardiovascular and cerebral events (MACCE) occur significantly more often in patients with atrial fibrillation, compared to patients without atrial fibrillation. Multivariate regression analysis confirmed atrial fibrillation (hazard ratio 2.39, 95%-confidence interval 1.06–5.41, p = 0.036) as an independent predictor for three-year-mortality after MitraClip implantation. *Conclusions*: Atrial fibrillation is an independent predictor for long-term mortality after MitraClip implantation. We demonstrate the association of atrial fibrillation with mortality and MACCE in the long-term follow-up of patients undergoing MitraClip implantation.

## 1. Introduction

Transcatheter edge-to-edge mitral valve repair by MitraClip (Abbott, Abbott Park, IL, USA) implantation has gained importance as alternative treatment option for high surgical risk patients with severe, symptomatic mitral regurgitation (MR). After its successful first-in-man experience in 2003 [1], the MitraClip implantation was soon challenged in the EVEREST trials primarily in patients with degenerative MR [1,2,3]. However, under real world conditions, the MitraClip system is nowadays widely used in patients with functional mitral regurgitation and concomitant cardiac comorbidities, such as atrial fibrillation [1].

Conversely, valvular heart disease is an independent predictor for the incidence of atrial fibrillation [4]. Regarding MR, in recent registries and trials, atrial fibrillation was found in 31.7–67.7% of all patients [2,5,6,7]. However, the impact of atrial fibrillation on the clinical outcome of patients treated with MitraClip remains to be determined.

In our study, we evaluate the impact of atrial fibrillation on clinical outcome in a three-year follow-up. We analysed 355 consecutive patients with and without pre-existing atrial fibrillation with severe MR and high surgical risk undergoing MitraClip implantation regarding mortality, major adverse cardiovascular and cerebral events (MACCE), rehospitalization due to heart failure and reintervention of the mitral valve.

## 2. Materials and Methods

### 2.1. Study Population

We evaluated 355 consecutive patients with high-grade MR who underwent MitraClip implantation between January 2010 and December 2016. No exclusion criteria existed for participation in our MiTraUlm registry. The interdisciplinary heart team made the decision for transcatheter edge-to-edge mitral valve repair by MitraClip due to inoperability or high surgical risk. The study complies with the principles of the Declaration of Helsinki and local regulations. The local ethics committee approved the research protocol. All patients provided written informed consent.

### 2.2. Baseline and Procedural and Post-Procedural Characteristics

All baseline, procedural and post-procedural data (symptoms, electrocardiograms, echocardiography, cardiac catheterization, blood testing) were prospectively recorded.

For further analysis, the study population of 355 patients undergoing MitraClip implantation was evaluated for history of atrial fibrillation (paroxysmal, persistent or permanent). All patients with a history of atrial fibrillation or evidence for atrial fibrillation in ECG or holter-ECG were allocated to the atrial fibrillation group (AFIB). 239 patients were allocated to the AFIB. 116 patients did not have a history of atrial fibrillation and absence of atrial fibrillation in available (current or prior) ECG and holter-ECG (no AFIB group). Procedural success was defined as successful delivery of the MitraClip device and the effective reduction of MR of more than two degrees.

Peri-procedural adverse events were recorded, including: in-hospital death, post-procedural myocardial infarction, major bleeding according to Mitral Valve Academic Research Consortium (MVARC) ≥ 2, cardiogenic shock, peri-procedural cardiopulmonary resuscitation (CPR) and stroke.

MR and measurement of mean transmitral pressure gradients by TTE was performed intra-procedural and one day after MitraClip implantation. Intra-procedural and post-procedural TTE was performed by cardiologists specialized for non-invasive imaging according to current recommendations of the European Society of Cardiology [8].

### 2.3. Clinical Follow-Up/Outcome

Follow-up was realized by clinical visit (62.1%) or phone contact (37.9%) up to three years after MitraClip implantation. Only one of 355 patients did not participate in follow-up visits. The clinical outcome was assessed including all-cause mortality, cardiovascular mortality, major adverse cardiovascular and cerebral events (MACCE), rehospitalization due to heart failure and reintervention of the mitral valve. MACCE was defined as death of any cause, rehospitalization due to heart failure, myocardial infarction, stroke (disabling and non-disabling) and major bleeding, reintervention of the mitral valve and the need for mechanical circulatory support.

### 2.4. Statistical Analysis

Pre-procedural parameters, procedural data and post-procedural outcomes were compared between two groups of patients: Patients with and without a history of atrial fibrillation. A value of p < 0.05 was considered statistically significant. All statistical analyses were performed using the Statistica software version 7.1 (Stat Soft, Inc., Tulsa, OK, USA) and medcalc Software 17.9.2 (MedCalc Software bvba, Ostend, Belgium).

Continuous, normally distributed variables are expressed as mean ± one standard deviation (SD) and were compared with Student t-test. Categorical variables are presented as frequencies and percentages. Differences between proportions were calculated by using Chi2 test. Time-to-event analyses for 36-month follow-up were performed with the use of Kaplan-Meier estimates and were compared with the log-rank test. Furthermore, we performed Kaplan-Meier survival analysis for time-to-event outcome in the 36-month follow-up for functional and degenerative aetiology of MR. Kaplan-Meier curves were generated for time-to-event outcomes regarding mortality, MACCE, rehospitalization due to heart failure and reintervention of the mitral valve in patients with and without atrial fibrillation.

Univariate Cox proportional-hazards regression analysis included all significant risk factors (p < 0.05 of Student t-test and Chi2 test). Multivariate Cox proportional-hazards regression analysis (full-model) was performed for all significant and probable variables (p < 0.1) of univariate analysis.

## 3. Results

We evaluated 355 consecutive patients with high-grade MR who underwent MitraClip implantation between January 2010 and December 2016. The study population was divided into two groups: One group consisting of 239 patients (67.3%) with atrial fibrillation (Afib) in the past medical history and 116 patients without atrial fibrillation (no Afib). 92 patients (38.5%) had paroxysmal Afib, 62 patients (25.9%) had persistent Afib and 85 patients (35.6%) had permanent Afib. Baseline characteristics of patients with and without atrial fibrillation are displayed in Table 1.

There were no statistically significant differences between patients with and without atrial fibrillation regarding comorbidities and preoperative surgical risk. However, in the patient group with atrial fibrillation (Afib group), the patients were older (77.6 ± 7.6 vs. 75.5 ± 9.8 years, p = 0.024) and had higher levels of baseline NT-pro-BNP (6517.5 ± 7308.0 vs. 4153.8 ± 4682.5 pg/mL; p = 0.010). Pre-procedural echocardiography (Table 2) revealed, that patients with atrial fibrillation had a larger left atrial diameter and more frequently severe tricuspid regurgitation. The procedural characteristics were equivalent in both groups. MitraClip implantation was successful in 98.7% and 96.6% respectively (Table 3) and post-procedural echocardiography showed similar results in both groups. The rate of in hospital adverse events did not significantly differ between patients with and without atrial fibrillation (Table 3). 11 patients died during hospitalization for MitraClip implantation. Two patients died of pneumonia (one in each group), three of stroke (two in the Afib group, one in the no Afib group), five of cardiogenic shock/low out-put failure (three in the Afib group, two in the no Afib group) and one patient of pulmonary embolism (in the Afib group).

The clinical outcome of both patient groups was evaluated in a three-year follow-up after MitraClip implantation. The median follow-up period was 389 days (interquartile range 202 days–746 days). Concomitant medication is listed in Table 4. The rate of anticoagulation remained unchanged throughout the three-year follow-up (72.6% (25.3% vitamin K antagonist, VKA + 47.3% direct oral anticoagulants, DOAC) at baseline and 72.4% (24.1% VKA + 49.3% DOAC) at three-year follow-up in the Afib group vs. 12.1% (5.2% VKA + 6.9% DOAC) and 13.3% (6.7% VKA + 6.7% DOAC) in the no Afib group). In addition, the use of anticoagulation was not different in the permanent vs. non-permanent atrial fibrillation groups (71.7% vs. 74.1%, p = 0.69). Hence, stroke rates during three-year-follow-up were low in both groups (6.4% vs. 4.9%, p = 0.72). Major bleeding occurred in 3.8% (N = 9) in the Afib group compared to 0.9% (N = 1) in the no Afib group (p = 0.09). Among the patients with major bleeding, one patient in the Afib group died 482 days after the bleeding event, thus likely unrelated to bleeding.

**Figure 1:**
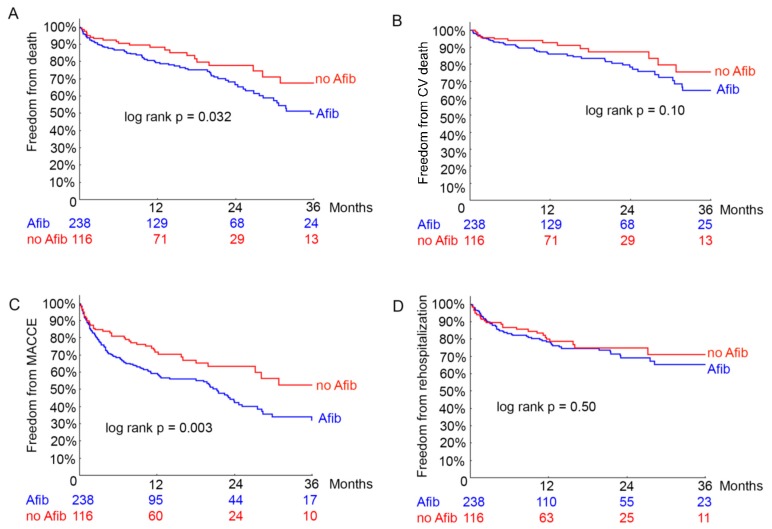
Long-term clinical outcome. Kaplan-Meier analysis for death (**A**), cardiovascular death (**B**), MACCE (**C**) and rehospitalization (**D**) in patients with atrial fibrillation (Afib) and without atrial fibrillation (no Afib).

Regarding all-cause mortality, in the atrial fibrillation group significantly more patients died during the three-year-follow-up after MitraClip implantation (50.3% vs. 32.2%, p = 0.032; Figure 1a, Table 5). The rate of cardiovascular death was higher in the atrial fibrillation group throughout the three-year follow-up after MitraClip implantation (35.1% vs. 24.2%, p = 0.10; Figure 1b, Table 5) without reaching statistical significance. MACCE occurred significantly more often in the atrial fibrillation group three-year-follow-up after MitraClip implantation (66.7% vs. 46.7%, p = 0.003; Figure 1c, Table 5). Rehospitalization for heart failure decompensation occurred more often in the Afib group, but did not reach statistical significance (p = 0.50, Figure 1d, Table 5). Likewise, NYHA functional class one year after MitraClip did not significantly differ between patients with and without atrial fibrillation (2.2 ± 0.9 vs. 2.2 ± 0.9, p = 0.66). The rate of reintervention of the mitral valve was low in both groups (1.5% vs. 0; p = 0.21).

**Figure 2:**
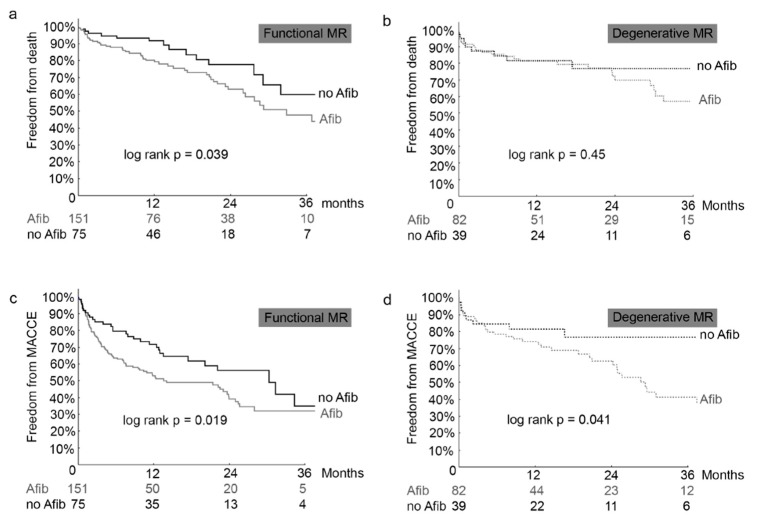
Forms of atrial fibrillation. Kaplan-Meier analysis for death (**a**), cardiovascular death (**b**), MACCE (**c**) and rehospitalization (**d**) in patients with atrial fibrillation (Afib) and without atrial fibrillation (no Afib).

Comparing the three forms of atrial fibrillation (paroxysmal and persistent vs. permanent), there was no significant difference of atrial fibrillation regarding mortality (p = 0.53), cardiovascular mortality (p = 0.93), MACCE (p = 0.48), and heart failure rehospitalization (p = 0.67, Figure 2).

Kaplan-Meier curves of all-cause mortality (A), cardiovascular (CV) mortality (B), MACCE (C), rehospitalization due to heart failure (D) of patients undergoing MitraClip implantation with paroxysmal, persistent or permanent Afib. par + pers = patients with either paroxysmal or persistent atrial fibrillation, perm = patients with permanent atrial fibrillation; MACCE = major adverse cardiovascular and cerebral events.

**Figure 3:**
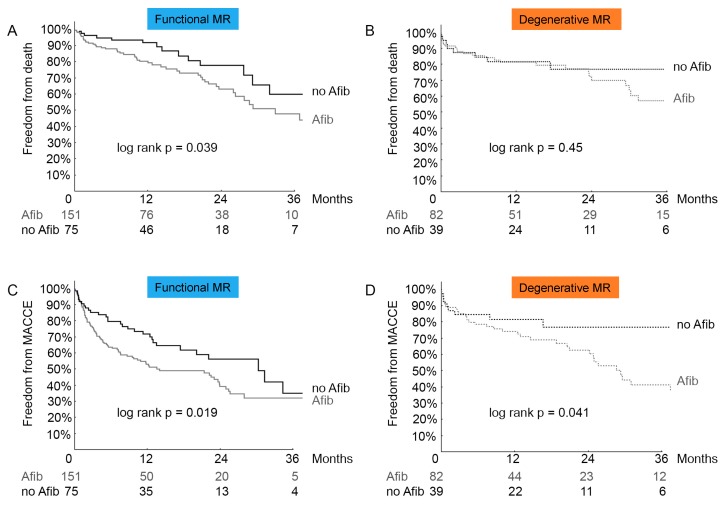
Functional and degenerative aetiologies of MR. Kaplan-Meier analysis for death (**A**), cardiovascular death (**B**), MACCE (**C**) and rehospitalization (**D**) in patients with atrial fibrillation (Afib) and without atrial fibrillation (no Afib).

Taking the different aetiologies of MR into account, we subdivided the study population into functional and degenerative MR. Regardless of the aetiology of MR, MACCE and mortality occurred consistently more often in the atrial fibrillation groups throughout 3-year-follow-up (Figure 3).

Kaplan-Meier curves of all-cause mortality (A functional, B degenerative), MACCE (C functional, D degenerative) of patients undergoing MitraClip implantation. Afib = patients with history of atrial fibrillation, MACCE = major adverse cardiovascular and cerebral events, no Afib = patients without atrial fibrillation.

Age, baseline NT-pro-BNP and Troponin T, left atrial diameter, pre-procedural high-grade tricuspid regurgitation, systolic pulmonary artery pressure, pulmonary capillary wedge pressure and atrial fibrillation were tested by univariate Cox proportional-hazards regression analysis. Baseline NT-pro-BNP (p = 0.046) and atrial fibrillation (p = 0.052) were identified as possible risk factors of three-year mortality and were included in further multivariate analysis. Left atrial diameter (p = 0.19), pre-procedural severe tricuspid regurgitation (p = 0.11), systolic pulmonary artery pressure (p = 0.32), pulmonary capillary wedge pressure (p = 0.40), baseline Troponin T (p = 0.67) and age (p = 0.49) failed to predict mortality after MitraClip implantation. Hence, multivariate Cox proportional-hazards regression analysis was performed for NT-pro-BNP and atrial fibrillation and identified history of atrial fibrillation (hazard ratio 2.39, 95% confidence interval 1.06–5.41; p = 0.036, Table 6) as independent predictor of mortality after adjustment for NT-pro-BNP (p = 0.072) levels in patients undergoing MitraClip implantation for severe MR.

## 4. Discussion

Valvular heart disease is an independent predictor for the incidence of atrial fibrillation [4]. Atrial fibrillation negatively impacts on the clinical outcome of patients with severe heart valve disease, valve surgery and transcatheter aortic valve implantation [9,10,11,12]. However, the impact of atrial fibrillation on the clinical outcomes of patients with MR undergoing MitraClip implantation has not been determined, yet. We evaluated 355 consecutive patients with severe MR and high surgical risk treated with transcatheter MitraClip implantation.

In recent trials and registries, atrial fibrillation was found in 31.7–67.7% of patients undergoing MitraClip implantation [2,5,6,7]. In our study, a history of atrial fibrillation was present in 67.3% of all patients. Hence, the impact of atrial fibrillation on clinical outcomes after MitraClip implantation is of great clinical relevance due to the high prevalence of atrial fibrillation in this population.

Regarding the baseline characteristics, our single-centre study population is comparable to the population of recent multi-centred trials and registries, thus accurately reflecting contemporary clinical practice.

In the long-term follow-up, we found all-cause mortality as well as MACCE to occur consistently more often in patients with pre-procedural atrial fibrillation compared to patients without atrial fibrillation throughout the three-year follow-up after MitraClip implantation. Furthermore, the form of atrial fibrillation (paroxysmal/persistent vs. permanent) had no impact on all-cause mortality, cardiovascular mortality, MACCE and rate of rehospitalization for heart failure decompensation. In consistency with the “real-world” registries ACESS-EU [6], TCVT [7] and TRAMI [5] registries, in our study population the aetiology of MR was mainly functional (62.5%). In these registries, clinical outcomes after MitraClip vary between the different aetiologies of MR [7]. However, in our study, atrial fibrillation is associated with increased three-year mortality and MACCE rate irrespective of the different aetiologies of MR.

In patients with a history of atrial fibrillation we found significantly higher rates of severe tricuspid regurgitation. Kammerlander et al. found, that patients with left heart valve procedure with severe tricuspid regurgitation had a higher likelihood of atrial fibrillation than those patients with non-significant tricuspid regurgitation, but was not associated with mortality [13]. Likewise, in univariate Cox regression analysis severe tricuspid regurgitation failed to predict three-year mortality in our cohort.

In the TRAMI registry [5] and in two smaller trials [14,15], NT-pro-BNP was found to correlate with long-term mortality in patients after MitraClip treatment. In the patient group with atrial fibrillation in our trial, NT-pro-BNP levels were elevated compared with patients without history of atrial fibrillation. Conversely, elevated NT-pro-BNP levels are commonly found in atrial fibrillation patients and may even be a predictor for the development of atrial fibrillation [4,16]. Thus, we included NT-pro-BNP in addition to atrial fibrillation in multivariate Cox regression analysis. Importantly, baseline NT-pro-BNP failed to predict three-year-mortality in our cohort. There are hints that NT-pro-BNP per se might not be an optimal predictor of clinical outcome in MitraClip patients [17]. This hypothesis is supported by the following facts: BNP is secreted belated from ventricular cardiomyocytes by gene expression whereas atrial cardiomyocytes secrete ANP instantly from secretory granules [18]. Hence, secretion of ANP occurs at subclinical stages, whereas BNP is secreted at end-stage. Furthermore, secretion of ANP is mediated by rising atrial pressures (e.g., in MR patients), whereas BNP levels are paralleled with ventricular pressures [19].

In patients undergoing mitral valve surgery, atrial fibrillation has been shown to adversely affect clinical outcomes in the long-term follow-up in some studies [11,12,20,21], whereas in others the outcomes were similar regardless of atrial fibrillation [22,23]. In recent registries of transcatheter MitraClip implantation, the rate of atrial fibrillation was higher in patients suffering post-procedural death [1,5,7,24,25]. In addition, Velu et al. found in 618 patients association of mortality with atrial fibrillation in a five-year follow-up, but atrial fibrillation failed to independently predict mortality in their registry [26]. Importantly, in this Dutch cohort, patients with and without atrial fibrillation differed with regard to prevalence of coronary artery disease and left ventricular function, demonstrating an inhomogeneous distribution of cardiac comorbidities before MitraClip implantation in this trial. In contrast, in our cohort, atrial fibrillation was not associated with advanced heart disease or different aetiologies of heart failure. Left ventricular ejection fraction, and cardiac index as well as pulmonary pressures were similar in patients with and without atrial fibrillation. Hence, this homogenous distribution of relevant cardiac comorbidities in both patient groups (with and without atrial fibrillation) in our cohort is of particular value for the appropriate analysis of the influence of atrial fibrillation on mortality after MitraClip implantation. In our trial, multivariate Cox proportional-hazards regression analysis confirmed pre-procedural atrial fibrillation as independent predictor of mortality with a hazard ratio of 2.4 after adjustment for NT-pro-BNP levels in patients undergoing MitraClip implantation for severe MR.

## 5. Conclusions

After MitraClip mortality and major adverse cardiovascular and cerebral events occur significantly more often in the three-year follow-up in patients with atrial fibrillation, compared to patients with sinus rhythm. Atrial fibrillation independently predicts long-term mortality after MitraClip implantation with a hazard ration of 2.4.

## Figures and Tables

**Table 1:** Baseline characteristics.

	Afib	No Afib	p-Value
Number of patients	239	116	
Age (years)	77.6 ± 7.6	75.5 ± 9.8	0.024
Female gender	90 (37.7%)	49 (42.2%)	0.41
BMI (kg/m^2^)	25.9 ± 4.3	25.4 ± 4.5	0.29
Diabetes mellitus	66 (27.6%)	38 (32.8%)	0.54
Creatinine (µmol/L)	131.9 ± 61.8	128.1 ± 79.8	0.63
Glomerular filtration rate (mL/min)	42.2 ± 15.8	46.3 ± 18.7	0.06
Hemoglobin (g/dL)	12.3 ± 1.8	12.2 ± 1.9	0.67
Troponin T (ng/L)	42.9 ± 43.9	76.9 ± 254.5	0.09
NT-pro-BNP (pg/mL)	6517.5 ± 7308.0	4153.8 ± 4682.5	0.010
Coronary artery disease	169 (71.1%)	89 (77.4%)	0.29
History of MI	52 (21.8%)	29 (25.0%)	0.33
History of cardiac bypass graft	37 (15.5%)	25 (21.6%)	0.16
Peripheral or cerebral vascular disease	63 (26.5%)	37 (32.5%)	0.25
History of stroke or intracerebral bleeding	25 (10.5%)	11 (9.7%)	0.80
Chronic obstructive pulmonary disease	34 (14.2%)	14 (12.1%)	0.57
ICD (pre-existing)	40 (16.7%)	24 (20.7%)	0.37
CRT (pre-existing)	19 (8.0%)	15 (12.9%)	0.14
NYHA class	3.2 ± 0.7	3.0 ± 0.8	0.16
EuroScore II	8.6 ± 7.7	8.9 ± 8.6	0.79
STS for mortality	4.2 ± 4.9	3.8 ± 4.1	0.37

Values are mean ± SD or n (%); BMI = Body mass index; Glomerular filtration rate (mL/min) according to chronic kidney disease epidemiology formula; MI = myocardial infarction; ICD = Implantable Cardiac Defibrillator; CRT = Cardiac Resynchronization Device; STS = Society of Thoracic Surgeons.

**Table 2:** Baseline echocardiographic and invasive parameters.

	Afib	No Afib	p-Value
Number of patients	239	116	
**Transthoracic echocardiography**			
Degenerative etiology of MR	82 (35.0%)	39 (34.2%)	0.50
MR grade (0–4)	3.5 ± 0.6	3.5 ± 0.5	0.36
LVEDD (mm)	60.5 ± 11.3	63.4 ± 11.5	0.06
LVESD (mm)	45.5 ± 14.0	49.2 ± 13.7	0.07
LA diameter (mm)	56.9 ± 10.1	51.6 ± 7.0	0.00002
Transvalvular mitral gradient (mmHg)	2.1 ± 2.1	1.8 ± 1.3	0.22
Left ventricular ejection fraction (%)	44.2 ± 18.0	42.3 ± 17.0	0.43
Tricuspid regurgitation grade III/IV	118 (56.2%)	31 (33.0%)	0.0002
**Cardiac catheterization**			
Cardiac output (L/min), Fick method	3.8 ± 1.2	3.7 ± 1.1	0.44
Cardiac index (L/min/m^2^)	2.0 ± 0.6	2.1 ± 0.5	0.77
Systolic pulmonary artery pressure (mmHg)	51.6 ± 15.9	47.8 ± 16.0	0.11
Mean pulmonary artery pressure (mmHg)	33.2 ± 10.6	31.3 ± 11.3	0.25
Pulmonary capillary wedge pressure (mmHg)	23.0 ± 9.1	20.2 ± 9.2	0.05
Pulmonary vascular resistance (dynxsxcm^−5^)	311 ± 270	285 ± 233	0.55

Values are mean ± SD or n (%). MR = Mitral Regurgitation; LVEDD = Left Ventricular End-Diastolic Diameter; LVESD = Left Ventricular End-Systolic Diameter; LA = Left Atrium.

**Table 3:** Procedural and post-procedural data.

	Afib	No Afib	p-Value
Number of patients	239	116	
**Implantation details**			
Procedural success	236 (98.7%)	112 (96.6%)	0.18
Number of clips	1.3 ± 0.5	1.3 ± 0.5	0.86
Clip detachment	5 (2.1%)	3 (2.5%)	0.78
**Echocardiography** **post-procedural**			
MR grade (0–4)	1.6 ± 0.6	1.6 ± 0.7	0.50
Transvalvular mitral gradient (mmHg)	4.0 ± 2.7	4.2 ± 2.2	0.70
**In-hospital outcome**			
ICU stay after procedure	1.3 ± 3.7	1.0 ± 1.6	0.80
Hospital stay after procedure	7.7 ± 5.7	7.2 ± 4.9	0.80
In-hospital death	7 (2.9%)	4 (3.5%)	0.79
Post-procedural myocardial infarction	0	0	n.a.
Cardiogenic shock	5 (2.1%)	1 (0.9%)	0.37
Need for CPR	4 (1.7%)	3 (2.6%)	0.57
Need for Inotropes	32 (13.5%)	13 (11.2%)	0.55
Stroke	4 (1.7%)	2 (1.7%)	0.97
Post-procedural bleeding MVARC ≥ 2	6 (2.5%)	2 (1.7%)	0.63
Infection (any)	9 (3.8%)	7 (6.0%)	0.34

Values are mean ± SD or n (%); MR = Mitral Regurgitation; ICU = Intensive Care Unit; CPR = Cardio-Pulmonary Resuscitation.

**Table 4:** Concomitant medication during three-year follow-up.

	Baseline			3 Months			12 Months			3 Years		
	Afib	No Afib	p-Value	Afib	No Afib	p-Value	Afib	No Afib	p-Value	Afib	No Afib	p-Value
Beta-blocker	92.8	91.2	0.60	92.6	87.5	0.23	90.0	89.6	0.93	79.3	93.3	0.20
ACE-I or ARB	78.9	85.7	0.12	75.0	80.3	0.39	70.5	79.2	0.11	62.1	86.7	0.08
MRA	41.0	46.4	0.34	46.3	50.7	0.55	39.2	40.3	0.88	44.8	46.7	0.91
ARNI	3.2	0	0.14	4.3	5.0	>0.89	5.4	4.3	0.77	n.a.	n.a.	

**Table 5:** Kaplan-Meier estimates and cumulative absolute numbers (in brackets) of three-year clinical outcome in patients with and without atrial fibrillation.

	12 Months		24 Months		36 Months		p-Value
	Afib	No Afib	Afib	No Afib	Afib	No Afib	
Mortality	(47) 21.1%	(13) 11.8%	(62) 34.0%	(19) 22.3%	(74) 50.3%	(22) 32.2%	0.032
Cardiovascular mortality	(29) 14.1%	(8) 7.5%	(37) 21.5%	(11) 13.0%	(45) 35.1%	(14) 24.2%	0.10
MACCE	(94) 41.5%	(31) 28.5%	(112) 57.6%	(36) 36.3%	(120) 66.7%	(39) 46.7%	0.003
Rehospitalization due to heart failure	(44) 21.9%	(21) 20.5%	(54) 31.0%	(24) 25.0%	(56) 34.6%	(25) 28.4%	0.50

Kaplan-Meier curves of all-cause mortality (A), cardiovascular (CV) mortality (B) MACCE (C) and rehospitalization due to heart failure (D) of patients undergoing MitraClip implantation. Afib = patients with history of atrial fibrillation, MACCE = major adverse cardiovascular and cerebral events, no Afib = patients without atrial fibrillation.

**Table 6:** Predictors of 3-year mortality after MitraClip implantation by multivariate Cox proportional-hazards regression analysis.

	Beta	Hazard Ratio	95% Confidence Interval	p-Value
Atrial fibrillation	0.872	2.39	1.06–5.41	0.036
NT-pro-BNP	0	1.0	1.0–1.0	0.072
